# Experience of patients with restraints in acute care hospitals and the view of their relatives: A qualitative study

**DOI:** 10.1002/nop2.1975

**Published:** 2023-08-23

**Authors:** Sandra Siegrist‐Dreier, Silvia Thomann, Isabelle Barbezat, Dirk Richter, Kai‐Uwe Schmitt, Sabine Hahn

**Affiliations:** ^1^ Bern University of Applied Sciences, School of Health Professions, Applied Research & Development in Nursing Bern Switzerland; ^2^ Department of Nursing, Academic‐Practice‐Partnership Bern University Hospital Bern Switzerland

**Keywords:** healthcare professionals, hospitals, nursing, patient experiences, qualitative research, relatives' experiences, restraint

## Abstract

**Aim:**

To describe the experiences of patients and relatives with any form of restraints in somatic acute care hospitals.

**Design:**

Qualitative explorative design.

**Methods:**

Qualitative research methods were used. Participants were recruited through clinical nursing specialists in participating departments of a university hospital between June and August 2020. Individual interviews were conducted and analysed using content analysis.

**Results:**

Four interviews with patients and five interviews with relatives were conducted with a mean duration of 25 min. The following three topics emerged in the analysis as important: *What was perceived as restraints, Assessing the experiences of restraint use on a continuum*, and *Lack of information about restrictive measures*. Patients and relatives defined restraint very broadly and assessed the experiences of restraint on a continuum from positive to negative, with a more critical view from patients. Relatives clearly seemed to approve of the use of restraints in acute care hospitals because it provided them with a sense of security. In general, there seemed to be a lack of information about the use of restraint and its effects on patients and relatives alike.

**Conclusion:**

The involvement of patients and relatives in the decision‐making process about restraint use seems to be low. Healthcare professionals need to be better educated to be able to pass on adequate information and to involve patients and their relatives adequately in all processes of restraint use. However, when relatives are involved in decision‐making as proxies for patients, it is important to consider that patients' and relatives' opinions on restraints may differ.

**Patient or Public Contribution:**

Patients and relatives agreed to participate in the study and shared their experiences with us.

## 1 INTRODUCTION

The EU Charter of Fundamental Rights (Article 3) emphasises that physical and mental integrity must be respected (European Union, 2012). Since restraining measures violate this high good of humanity, they may only be used as a last resort. Nevertheless, restraints are currently used in all areas of healthcare (Barbui et al., 2021; Gunawardena & Smithard, 2019; Scheepmans et al., 2020). In mental health and long‐term care, the use of restraining measures has been the focus of numerous studies and initiatives aimed at reducing these procedures and improving the safety of services for patients and staff alike (Cusack et al., 2018; Freeman et al., 2016; Scheepmans et al., 2020). In recent years, the issue has also been increasingly studied in somatic (non‐psychiatric) acute care hospitals (Abraham et al., 2020).

In somatic acute care hospitals, the different types of restraints include physical (mechanical) restraints such as belts, bed rails, fixed chairs or being held down by healthcare professionals (Bleijlevens et al., 2016); chemical (pharmacological) restraints that include all sedative medications administered for the sole purpose of preventing violent agitation or other disruptive behaviour; and environmental restraints such as locked wards, one‐to‐one supervision and electronic measures (cameras, sensor mats, trackers, etc.) (Registered Nurses' Association of Ontario [RNAO], 2012). The prevalence of restraints in acute care hospitals varies greatly depending on the definition and legal regulations (Krüger et al., 2013; Martin & Mathisen, 2005), but can affect up to 44.5% of patients (Nakanishi et al., 2018; Spennato et al., 2023; Thomann et al., 2021).

In acute care hospitals, restraints are most often justified as a security measure to prevent confused or agitated patients from endangering themselves or others (Ang et al., 2015; Evans et al., 2003; Thomann et al., 2021). However, restraints have not been shown to have the desired effect, for example, preventing falls or the accidental removal of catheters and tubes (Chao et al., 2017; LeLaurin & Shorr, 2019; Perez et al., 2019). In contrast, there is evidence of significant physical and psychological health risks associated with physical and chemical restraints (Evans et al., 2003; Gunawardena & Smithard, 2019; RNAO, 2012; Warlan & Howland, 2015). Existing studies in acute care hospitals have mainly investigated the prevalence of restraints and the possible effects of different restraining measures (Agrawal et al., 2012; Ang et al., 2015), as well as the views and experiences of healthcare professionals (Canzan et al., 2021; Li & Fawcett, 2014; Siegrist‐Dreier et al., 2022). Although it is in fact ethically and legally required to act according to the presumed will of the patient (American Medical Association, n.d.; Swiss Academy of Medical Sciences, 2015), only a few studies have addressed what is experienced by patients. These studies show that patients perceive restraint use negatively (Gallinagh et al., 2001; Wong et al., 2020). Relatives often act as the patients' legal representatives and are interviewed by proxy when patients are no longer able to provide information. They therefore also represent an important group. Since the perspectives of affected patients and their relatives may differ from those of healthcare professionals, it is important to give these groups a voice and include them in the decision‐making process (Jacob et al., 2019). Ethical and legal guidelines mandate that relatives must be informed about the measures, or must agree to them by proxy (American Medical Association, n.d.; Australian Government Aged Care Quality and Safety Commission, 2020). In addition, it is of great importance to raise awareness among healthcare professionals of the patients' perspective, thus ensuring that patients are more involved in the decision‐making process. Therefore, the aim of this study was to describe how patients and relatives experienced restraint use in the somatic acute care hospital.

## 2 METHODS

An explorative descriptive qualitative design was used to investigate the research aim. With this open approach, a broad view of the topic was allowed, without limiting possible outcomes from the outset, as is recommended for a subject on which little is known to date (Gray et al., 2017). The COREQ guideline was used for reporting (Tong et al., 2007).

### 2.1 Sample and recruitment

The study was conducted in a 900‐bed university hospital in Switzerland between Mai and August 2020. At the participating university hospital, internal analyses identified several departments where restraints were known to be used. The departments of Internal Medicine and Neurology were included in the study. No patients were recruited in intensive care units because the patients there would usually have been too ill to obtain informed consent. However, the included patients and relatives talked about their experiences during the whole hospital stay and therefore some of them also remembered experiences in the intensive care units. The recruitment was done by clinical nursing specialists from the participating departments. They handed out study information and asked patients with good cognitive health who had experienced any form of restraints, as well as their relatives, to participate in the study. No other inclusion or exclusion criteria were applied. Patients and relatives who agreed to participate were registered, and a member of the research team was informed that they had consented to initial phone contact to schedule an interview.

### 2.2 Data collection

On initial contact, study participants could choose whether they wanted to be interviewed by telephone or face‐to‐face. The interviews, conducted by the first author, opened with an introductory question that invited participants to speak freely about their experiences with restraint. Participants were asked to describe their experiences in their own words and to address the issues that were of importance to them, without topics being given. The authors had collectively created a guideline with follow‐up questions that were asked if needed to keep the conversation going and that served as orientation but that were not used as a rigid structure. The questions in the guideline were developed based on the literature (mentioned in the introduction). They revolved around experiences of restraints, feelings in connection with restraints, memories, positive and negative effects, information given by the health care team and wishes towards this team, processes in connection with restraints, etc. The first interview served as a pre‐test. Afterwards, the study team discussed whether the guideline contained the necessary content, which was confirmed. Since no changes were necessary to the guideline, the first interview was also included in the study. The first author, a Master educated nurse with professional experience in intensive care, conducted the interviews. The interview guideline in its original language can be requested from the study team. Supplementary notes on the interviews were recorded in a research diary. To comply with the privacy of data standards, no further sociodemographic data were collected.

### 2.3 Data analysis

The interviews were digitally recorded by the first author, and anonymised following the rule‐guided method of transcription of Kuckartz (2008). Qualitative content analysis was used to analyse the data, with which patterns and recurring ideas can be extracted from text using an explorative, qualitative design (Gray et al., 2017). The program MAXQDA 2020 (VERBI Software, 2019) was used for the analysis. Since there was little prior knowledge about the topic, an inductive analysis was carried out based on the analysis process of Elo and Kyngäs (2008). The interviews of the patients and those of the relatives were processed together. The statements used were recorded in a way that made it clear from which perspective they were reporting. The analysis was performed by the first author and the last author, an expert in qualitative research and the subject of restrictive and coercive measures used in health care. It consisted of three phases. In the *preparation phase*, the transcribed text was repeatedly read independently by the two authors to familiarise themselves with the content. The subsequent *organising phase* began with an independent open coding of the statements provided in the text by the first author. The categories and subcategories were conceptualised and defined in an inductive process. For this purpose, the data material was not only looked at several times, whereby characteristics of interviews, but also similar concepts across interviews could be identified. The categories and subcategories were critically discussed between the first and last authors. The content of all interviews was categorised to form the main categories. In the *resulting phase*, categories representing the research topics were formed and named according to their content in collaboration with all authors of the study team to ensure intersubjectivity.

### 2.4 Ethical considerations

The study protocol was submitted to the responsible ethics committee, which declared that the study does not fall under the Swiss Human Research Act (BASEC‐Nr. Req‐2019‐0025). Before the start of the interviews, participants were informed about the digital recording. They were asked again if they consented to participate in the study. It was pointed out that they were allowed to stop the interview at any time without consequences and that they were free to decide about which topics they wanted to provide information. They signed an informed consent form when they agreed to participate.

## 3 RESULTS

Four patients who had experienced restraints and five relatives of patients who had been restrained were interviewed between June and August 2020. One of these interviews was conducted together with the patient concerned and his relatives, resulting in a total of eight interviews. Five interviews took place over the phone and three at the participants' homes. They lasted between 15 min and 1 h (mean duration 25 min). Please refer Tables 1 and 2.

**TABLE 1:** Overview of the study participants (patients).

Participant	Gender	Restraints mentioned in the interview
Patient 1	Male	Sensor mats, sedatives
Patient 2	Male	Sensor mats, bed rails
Patient 3	Female	One‐to‐one supervision, sedatives
Patient 4	Male	Sensor mats, one‐to‐one supervision, motion sensors, sedatives

**TABLE 2:** Overview of the study participants (relatives).

Participant	Gender	Relation of relatives to patients with restraints	Restraints mentioned in the interview
Relative 1	Female	Spouse	Bed rails, sensor mats, sedatives
Relative 2	Female	Spouse	Sensor mats, motion sensors, fixing tables in front of armchairs and wheelchairs, locked windows/doors, bed rails
Relative 3	Female	Parent	Sensor mats, bed rails
Relative 4	Male	Son	Sensor mats, bed rail, mechanical fixation of hands
Relative 5	Male	Spouse	One‐to‐one supervision, mechanical fixation of hands, mittens, fixing tables in front of armchairs and wheelchairs

Most patients participating in this study or described by their relatives taking part experienced some memory difficulties. These had various causes, including accident with coma, severe injuries requiring analgesia, delirium and dementia. This affected their ability to report their experiences and may have influenced the narratives.

The following three topics emerged in the analysis as important from the viewpoint of patients and their relatives: *What was perceived as restraints*, *Assessing the experiences of restraint use on a continuum*, and *Lack of information about restrictive measures*.

### 3.1 What was perceived as restraints

Both the patients and relatives described restraints that matched the general definition given in the introduction of this article. However, their descriptions also went beyond this definition, seeing the concept of restraints in a broader way. One patient described her own disabling injuries as restraining. A relative also defined restraint as “the restrictions imposed by the disease” (Relative no. 3). Before proceeding with the interviews, a common understanding of restraint was established so that statements about experiences would refer only to the forementioned definition of restraint. Patients expressed that they considered the sensor mat placed next to the bed to be restraining, “because I am more or less monitored by it” (Patient no. 1). Medication to impair consciousness (sedatives), motion sensors and one‐to‐one supervision were other restraints that the patients had experienced. In their descriptions of what counts as a restraint, they also listed physical fixations, but had not experienced these themselves. The relatives had witnessed the mechanical fixation of hands, fixing tables in front of armchairs and wheelchairs, mittens, sensor mats, bed rails and locked windows/doors.

One patient distinguished between obvious and hidden restraints. He defined the latter as, for example, medications that continued to be administered even though they were no longer necessary, or therapies that were carried out without explanation. One patient described “everything between being forbidden to stand up alone and being tied up” (Patient no. 4) as restraint. However, not all restraints were recognised as such by relatives:

They [the nurses] attached blankets to the bed rails so that he [the patient] would not fall out of bed or hurt himself during a seizure. That does not count as a restraint then. (Relative no. 4)

In general, patients described the use of restraints in more emotional terms, whereas the relatives remained more factual.

### 3.2 Assessing the experience of restraint use on a continuum

Perceptions of the experiences of restraint use in the acute care hospital by patients and relatives ranged across a continuum, from considering it to be something positive that was described in non‐critical words, to being neutral, to viewing it as something negative described with critical words. Besides the different perceptions, there were also different explanatory patterns that patients and relatives used to account for the use of restraints, such as disease‐specific condition, environmental circumstances or the overall situation.

On the positive side of the continuum, some of the patients and relatives were understanding and supportive of the use of restraint, because they believed that there was a need for it for reasons of safety or protection. “I needed these strong medications to be able to lie still at all. And I was not allowed to move because of my severe injuries.” (Patient no. 3). “For me, it's a relief to know that they put the sensor mat on the floor for him, that I know when he gets up, someone will go and check on him.” (Relative no. 2). The relatives also attributed restraint use to the need to protect monitor wires and invasive devices as well as to prevent falls. As an environmental factor for restraint use, the relatives described that there were insufficient staff to be at the bedside of every agitated patient.

Relatives who explained measures due to disease‐specific conditions also rated restraint use on the positive side of the continuum. They reported that patients had epileptic seizures, for example, and therefore bed rails were attached. Other patients were impaired in their judgement due to dementia or delirium and, according to their relatives, could have endangered themselves without being aware of it. Additionally, some of the relatives said that situation‐specific circumstances required the use of restraints, and therefore regarded them to be on the positive side of the continuum.

In the intensive care unit, they [the nurses] put her [the patient] in a rubber chair [on the edge of the bed]. They had to tie her to it because she was tipping forward. (Relative no. 5)

A final explanatory pattern for ranking restraint use on the positive side of the continuum was characterised by a high degree of trust in the staff by relatives, some of whom explicitly said that they did not question the decisions of the professionals as they had a lot of experience.

I don't think we [the relatives] need to interfere much. If they [the healthcare professionals] have the experience to do something [restraints], I think it's good if they go through with it. It's better to do it once too often than too rarely […]. You can't stand next to every patient and see whether he wants to get up or not. That is […] impossible for the staff. (Relative no. 3)

Some restraint use was rated neutrally on the spectrum by patients. For example, one‐to‐one supervision by a sitter was not experienced as restrictive or disturbing; the participants never even thought about why the sitters were there. One patient perceived them as “extra‐human” beings who just sat there without communicating with him, but he was not frightened by them.

At the other end of the perception spectrum stood the view of patients who were bothered by restraint use and rated it negatively. A feeling of insecurity and experiencing the restrictive measures as disturbing led to this negative perception. A patient who had a sensor mat in front of the bed expressed “It's very unpleasant when an alarm goes off every time I get up or when I go out for a smoke. […] I can't lie in bed all day and I want to be as mobile as possible. That is important.” (Patient no. 1). These patients did not share the healthcare professionals' opinion that they were at risk of falling. They did not let the sensor mats stop them and got up on their own when necessary. One patient said that it was very difficult to talk about his restraints during rounds, as he shared a room with other patients, and they could overhear the conversation. All the patients said that the idea of being strapped down was frightening.

The relatives did not express any negative views about restraint use from their perspective, because, in their eyes, restraint use always served a purpose. However, they sometimes noticed that the patients were bothered by the measures as they could not understand their meaning. “I watched her trying to get rid of these mittens by just biting into them with her teeth and tearing the mitten off her hand. She was able to take the mitten off about three or four times […]” (Relative no. 5). Some relatives stated that they could understand that patients would find it demeaning to be monitored. Acceptance of restraint use was described as a process for both the patients and the relatives. One relative reported that the patient did not want to be a burden to anyone and therefore found it uncomfortable that she was not allowed to get up on her own, for instance.

**FIGURE 1:**
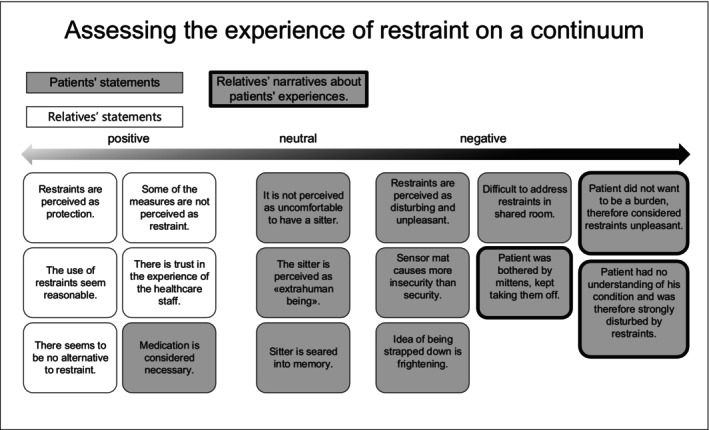
Summary of the different topics and their positions on the continuum.

Figure 1 shows a summary of the different topics and their positions on the continuum.

### 3.3 Lack of information about restrictive measures

The patients and their relatives said that they were not informed in advance about the use of restraints. Most of them received information about the purpose of the measures during their use. A few relatives had to ask why certain measures had been put in place in order to get information about them. Some patients figured this out for themselves. “[…] The sensor mat was just installed. I then noticed what it was doing because every time I got up someone came in.” (Patient no. 1). Some patients said that they did not remember receiving information, but that this could also be due to their impaired perception at the time. When information was provided, patients and relatives were informed about what benefits were expected from restraints; possible disadvantages or dangers seem not to have been addressed. Only a few patients or relatives were explicitly informed about the termination of the restraints and there was no debriefing for anyone. However, the patients and relatives interviewed stated that they did not feel the need for a debriefing.

The relatives reported the absence of information on another point: Sometimes it was very difficult for them to understand why restraints were no longer in place or were downgraded. They feared for the safety of the patients if, in their eyes, nothing had changed in their condition, but the restraints were no longer being implemented.

In the beginning, she was on a ward […] where she had one‐to‐one supervision 24 hours a day. Then on the other ward 50 meters away, that was not possible anymore. That was extremely stressful for me. […] But she had a sensor mat […] and they told her to call when she wanted to get up. […] In the end, nothing happened. (Relative no. 5)

The relatives said that they could not imagine any alternative to restraints. However, they observed preventive measures used in the hospital to prevent restraints or reduce them more quickly. Relatives witnessed that invasive catheters were removed as soon as possible because the restraints were necessary primarily to protect the catheters. Another relative reported that patients were given an orientation by means of information signs in the room or on toilet doors to reduce or prevent confusion.

## 4 DISCUSSION

The aim of this study was to describe the experiences with restraint use of patients in the acute care hospital and their relatives. Our analysis showed that patients and relatives define restraint very broadly and assess the experience of restraint on a continuum from positive to negative, with a more critical view from patients. However, patients said that their memory was partially or severely clouded in connection with the restraint use and that they could not adequately assess everything that happened in retrospect. Relatives clearly seemed to approve of the use of restraints in acute care hospitals because it provided them with a sense of security. They showed great trust in the healthcare professionals and assumed that the staff use their expert knowledge to make the right decisions. In general, among patients and relatives alike, there seemed to be a great lack of information about restraint and its effects, both in general and in their specific situation.

Our interviews revealed that the extent to which a restraint measure is experienced as restrictive seems to be very individual. Electronic restraints, such as sensor mats or light barriers, were described by some patients as unpleasant and disturbing. The review by Evans and Fitzgerald (2002), which included only physical restraints, described restraints as being perceived not only as a restriction of movement but also as a loss of freedom and an experience of being controlled. These limitations on freedom and loss of control also seemed to be experienced by the patients participating in our study during non‐physical restraints.

In previous studies, electronic restraints in the form of sensor alarms for beds and chairs were recommended as a means of reducing physical restraints and were perceived as an improvement by healthcare professionals (Evans & Fitzgerald, 2002; Gallinagh et al., 2001). This is actually in line with ethical and legal requirements, which mandate that healthcare professionals are obliged to choose the least distressing measure for patients when restraint is inevitable (American Medical Association, n.d.; Australian Government Aged Care Quality and Safety Commission, 2020). However, our results clearly show that electronic restraints can also be perceived as restricting freedom and thus, as recent guidelines on restraints already state (Swiss Academy of Medical Sciences, 2015), must be implemented with regard to ethical‐legal requirements. This means, among other things, that before the installation of these restraints, it must be ensured that a patient is incapable of judgement, that the relatives have been informed, that the restraint has more advantages than disadvantages, and that it is documented and evaluated (American Medical Association, n.d.; Australian Government Aged Care Quality and Safety Commission, 2020). In contrast to electronic measures, participants in our study did not perceive one‐to‐one supervision by a sitter as intrusive.

The results of our study point to potential difficulties in implementing these ethical‐legal requirements in acute care hospitals. For instance, patients and relatives were not sufficiently informed about restraints, especially regarding possible disadvantages and risks. This leads to an inability to make an informed decision about the usefulness of restraints and, therefore, results in too little involvement in the decision‐making process. In this context, it is quite remarkable that relatives expressed unprejudiced trust in healthcare professionals, as they were frequently not informed promptly or at all about the start of a restraint, usually only receiving information about the restraints when they came to visit. Nevertheless, all relatives interviewed stated that this did not bother them. However, it should also be mentioned that healthcare professionals are legally and ethically obliged to provide prompt information, even though the relatives in our study did not report any issues with its lack of provision (American Medical Association, n.d.; Australian Government Aged Care Quality and Safety Commission, 2020).

In long‐term care, it has been shown that the indication for a restraint is considered more carefully if the measures have to be justified, be it to relatives or in a reporting system (Konetzka et al., 2014). Additionally, it is known from the acute care setting that documentation of restraint is often inadequate (Thomann et al., 2021). Both points need to be improved so that restraints are reduced and used only when necessary. However, there is evidence in the literature that healthcare professionals generally tend to be insufficiently informed about the harms and benefits of restraints, and about what legally and ethically needs to be considered before employing them. Scientific findings, such as the lack of evidence on the effectiveness of restraints while their potential harm is proven, do not seem to sufficiently find their way into hospitals (Canzan et al., 2021; Siegrist‐Dreier et al., 2022), and therefore adequate information is not reaching patients and relatives. Other ethical‐legal recommendations also appear to be little known or implemented, particularly for restraints other than fixation belts (Thomann et al., 2022).

Despite the recommendations, it should not be forgotten that restraint use can be perceived as a source of stigma (Evans & Fitzgerald, 2002). Our study showed that it can be difficult for patients to talk about their restraints in the presence of other patients. This may also have led to patients being reluctant to ask for information or ask to be actively involved in the decision‐making or evaluation process. This clearly indicates that restraint use is a highly sensitive issue and that environmental factors also play a role and need to be taken into account. The extent to which it is permissible and appropriate to use any form of restraint in a shared room should be investigated. It is conceivable that there may also be advantages to being in a shared room for patients, as there is more supervision, but this is clearly a delicate issue and needs to be explored further.

In addition, our results point to another aspect relevant to the ethical‐legal recommendations for decision‐making: Patients and relatives perceived restraints very differently. In general, the relatives in our study consistently viewed restraint as something necessary and useful, while patients viewed them more critically. Relatives were informed by healthcare professionals about the restraints and did not question them. They listened to and accepted the justifications of the healthcare professionals, which is also reflected in their rather matter‐of‐fact narrative. This is highly relevant given that relatives are often involved in the decision‐making process on behalf of the patient, and it raises the question of whether this approach is adequate. In long‐term care, for example, it is recommended that restraint use is independently reviewed by a team of experts (Bellenger et al., 2019). This approach could also be considered in acute care hospitals. On the other hand, it should be noted that our findings contradict those of the review by Evans and Fitzgerald (2002), which described restraints as a source of anger and discomfort for relatives, with only a few relatives and patients considering them to be an effective safety measure. However, this review focused exclusively on physical restraints. Overall, this indicates an urgent need for a differentiated investigation of this topic.

In general, information on the significance of proxy opinion‐seeking for patients in the acute hospital setting is scarce, particularly in the context of restraints (Devnani et al., 2017). Nevertheless, the diversity of perceptions underscores the importance of talking about this sensitive issue with the people directly affected. The different opinions of patients and relatives, as well as the fact that not enough in‐depth information about restraint was available for relatives, need to be considered when obtaining a proxy opinion for patients. In general, our results highlight the importance of empathetically considering what it may mean for the patient when using a restraint, as has already been recommended by other authors (Gallinagh et al., 2001; Thomann et al., 2022).

### 4.1 Limitations

Since very few studies of patients and relatives on the experience of restraints in acute care hospitals are available, exceptionally old references had to be used in this study. In talking to the patients, it became apparent that many patients with whom restraints are used only have vague memories of the hospital stay or the restraints, which can influence their experiences and narratives. Furthermore, recruitment bias cannot be ruled out, as participants were approached and reported by clinical nursing specialists of the participating hospital. Since recruitment of participants proved extremely difficult, it was not possible to ensure that data saturation was achieved, which also limits generalizability. The first author has experience with the use of restraints from her professional life, which prevents a completely unbiased attitude from being adopted during data collection. On the contrary, the proximity to the subject matter allowed for a comprehensive understanding of the complex contexts related to restraints.

## 5 CONCLUSION

Our study shows that restraints are perceived very individually and that electronic measures such as sensor mats and light barriers can also be experienced as restrictive. Whenever possible, the patient or relatives should be involved in the decision‐making process if restraint is inevitable, so that the patient and their needs are at the centre of considerations. It is of great importance that the ethical‐legal guidelines in place are known and taken into account. This includes providing patients or relatives with detailed information, not only about the expected benefits of restraint, but also about its possible disadvantages and risks. In order to ensure this, healthcare professionals themselves need to be better trained and more knowledgeable in the field of ethical‐legal recommendations for restraints. When the obligation to provide information to patients and relatives is better fulfilled, further consideration about restraints might be undertaken, so that they are used less often and in a more focused way.

However, healthcare professionals need to be aware that, while it is both helpful and important to consult with relatives by proxy when patients are unable to express their own views, these views may well differ from those of the patients. Therefore, the patient's opinion should always be obtained as soon as this is possible again. If patients disagree with the restraints, this must be taken seriously. Healthcare professionals and relatives should always mentally put themselves in the patient's shoes in order to deepen their understanding of the situation.

It is important that further research be conducted with patients who are directly affected in order to map their perceptions and views more clearly. In addition, the largely unexplored issue of proxy decision‐making by relatives with potentially divergent perspectives needs to be further investigated.

## AUTHOR CONTRIBUTIONS

All the listed authors meet the authorship criteria, and all authors agree with the content of the manuscript. All authors designed the study. SSD collected the data. SSD and SH analysed the data and adapted the results after in‐depth discussions with all authors. SSD and SH initially prepared the manuscript, which was subsequently revised by all authors. All the authors approved the final version for submission.

## CONFLICT OF INTEREST STATEMENT

The authors declare no conflict of interest.

## REFERENCES

More than 25 references were needed to support the accuracy of the statements made. Some references are older than 5 years and had to be used because their important statements were not reproduced in more recent references.

## Data Availability

Requests for data shall be addressed to the corresponding author.

## References

[nop21975-bib-0001] Abraham, J. , Hirt, J. , Kamm, F. , & Möhler, R. (2020). Interventions to reduce physical restraints in general hospital settings: A scoping review of components and characteristics. Journal of Clinical Nursing, 29(17–18), 3183–3200. 10.1111/jocn.15381 32558091

[nop21975-bib-0002] Agrawal, V. , Mehta, R. , Dave, K. , & Shah, N. (2012). Physical and chemical restraint practices among nursing and medical staff in medical, surgical and psychiatric wards in a general hospital setting [conference abstract]. Indian Journal of Psychiatry, 54, S46 http://www.embase.com/search/results?subaction=viewrecord&from=export&id=L70971891

[nop21975-bib-0003] American Medical Association . (n.d.). Use of restraints . https://www.ama‐assn.org/delivering‐care/ethics/use‐restraints

[nop21975-bib-0004] Ang, S. Y. , Bakar Aloweni, F. A. , Perera, K. , Wee, S. L. , Manickam, A. , Lee, J. H. M. , Haridas, D. , Shamsudin, H. F. , & Chan, J. K. (2015). Physical restraints among the elderly in the acute care setting: Prevalence, complications and its association with patients' characteristics. Proceedings of Singapore Healthcare, 24(3), 137–143. 10.1177/2010105815596092

[nop21975-bib-0005] Australian Government Aged Care Quality and Safety Commission . (2020). Minimising the use of restraints . Retrieved September 30, 2020, from https://www.agedcarequality.gov.au/providers/assessment‐processes/minimising‐restraints

[nop21975-bib-0006] Barbui, C. , Purgato, M. , Abdulmalik, J. , Caldas‐de‐Almeida, J. M. , Eaton, J. , Gureje, O. , Hanlon, C. , Nosè, M. , Ostuzzi, G. , Saraceno, B. , Saxena, S. , Tedeschi, F. , & Thornicroft, G. (2021). Efficacy of interventions to reduce coercive treatment in mental health services: Umbrella review of randomised evidence. The British Journal of Psychiatry, 218(4), 185–195. 10.1192/bjp.2020.144 32847633

[nop21975-bib-0007] Bellenger, E. N. , Ibrahim, J. E. , Kennedy, B. , & Bugeja, L. (2019). Prevention of physical restraint use among nursing home residents in Australia: The top three recommendations from experts and stakeholders. International Journal of Older People Nursing, 14(1), e12218. 10.1111/opn.12218 30609220

[nop21975-bib-0040] Bleijlevens, M. H. C. , Wagner, L. M. , Capezuti, E. , Hamers, J. P. H. , & International Physical Restraint Workgroup (2016). Physical restraints: Consensus of a research definition using a modified delphi technique. Journal of the American Geriatrics Society, 64(11), 2307–2310. 10.1111/jgs.14435 27640335

[nop21975-bib-0008] Canzan, F. , Mezzalira, E. , Solato, G. , Mortari, L. , Brugnolli, A. , Saiani, L. , Debiasi, M. , & Ambrosi, E. (2021). Nurses' views on the use of physical restraints in intensive care: A qualitative study. International Journal of Environmental Research and Public Health, 18(18), 9646. 10.3390/ijerph18189646 34574571 PMC8464991

[nop21975-bib-0009] Chao, C. M. , Lai, C. C. , Chan, K. S. , Cheng, K. C. , Ho, C. H. , Chen, C. M. , & Chou, W. (2017). Multidisciplinary interventions and continuous quality improvement to reduce unplanned extubation in adult intensive care units: A 15‐year experience. Medicine (Baltimore), 96(27), e6877. 10.1097/md.0000000000006877 28682859 PMC5502132

[nop21975-bib-0010] Cusack, P. , Cusack, F. P. , McAndrew, S. , McKeown, M. , & Duxbury, J. (2018). An integrative review exploring the physical and psychological harm inherent in using restraint in mental health inpatient settings. International Journal of Mental Health Nursing, 27(3), 1162–1176. 10.1111/inm.12432 29352514

[nop21975-bib-0011] Devnani, R. , Slaven, J. E., Jr. , Bosslet, G. T. , Montz, K. , Inger, L. , Burke, E. S. , & Torke, A. M. (2017). How surrogates decide: A secondary data analysis of decision‐making principles used by the surrogates of hospitalized older adults. Journal of General Internal Medicine, 32(12), 1285–1293. 10.1007/s11606-017-4158-z 28840485 PMC5698224

[nop21975-bib-0012] Elo, S. , & Kyngäs, H. (2008). The qualitative content analysis process. Journal of Advanced Nursing, 62(1), 107–115. 10.1111/j.1365-2648.2007.04569.x 18352969

[nop21975-bib-0013] European Union . (2012). Charter of fundamental rights of the european union . http://data.europa.eu/eli/treaty/char_2012/oj

[nop21975-bib-0014] Evans, D. , & Fitzgerald, M. (2002). The experience of physical restraint: A systematic review of qualitative research. Contemporary Nurse, 13(2–3), 126–135. 10.5172/conu.13.2-3.126 16116768

[nop21975-bib-0015] Evans, D. , Wood, J. , & Lambert, L. (2003). Patient injury and physical restraint devices: A systematic review. Journal of Advanced Nursing, 41(3), 274–282. 10.1046/j.1365-2648.2003.02501.x 12581115

[nop21975-bib-0016] Freeman, S. , Hallett, C. , & McHugh, G. (2016). Physical restraint: Experiences, attitudes and opinions of adult intensive care unit nurses. Nursing in Critical Care, 21(2), 78–87. 10.1111/nicc.12197 26219511

[nop21975-bib-0017] Gallinagh, R. , Nevin, R. , McAleese, L. , & Campbell, L. (2001). Perceptions of older people who have experienced physical restraint. British Journal of Nursing, 10(13), 852–859. 10.12968/bjon.2001.10.13.852 11927885

[nop21975-bib-0018] Gray, J. , Grove, S. K. , & Sutherland, S. (2017). Burns and Grove's the practice of nursing research: Appraisal, synthesis, and generation of evidence. Elsevier.

[nop21975-bib-0019] Gunawardena, R. , & Smithard, D. G. (2019). The attitudes towards the use of restraint and restrictive intervention amongst healthcare staff on acute medical and frailty wards‐A brief literature review. Geriatrics (Basel), 4(3), 50. 10.3390/geriatrics4030050 31487923 PMC6787583

[nop21975-bib-0020] Jacob, J. D. , Holmes, D. , Rioux, D. , Corneau, P. , & MacPhee, C. (2019). Convergence and divergence: An analysis of mechanical restraints. Nursing Ethics, 26(4), 1009–1026. 10.1177/0969733017736923 29129122

[nop21975-bib-0021] Konetzka, R. T. , Brauner, D. J. , Shega, J. , & Werner, R. M. (2014). The effects of public reporting on physical restraints and antipsychotic use in nursing home residents with severe cognitive impairment. Journal of the American Geriatrics Society, 62(3), 454–461. 10.1111/jgs.12711 24617991 PMC6345266

[nop21975-bib-0022] Krüger, C. , Mayer, H. , Haastert, B. , & Meyer, G. (2013). Use of physical restraints in acute hospitals in Germany: A multi‐Centre cross‐sectional study. International Journal of Nursing Studies, 50(12), 1599–1606. 10.1016/j.ijnurstu.2013.05.005 23768409

[nop21975-bib-0023] Kuckartz, U. (2008). Qualitative evaluation der einstieg in die praxis (2., aktualisierte Aufl. ed.). VS Verlag für Sozialwissenschaften.

[nop21975-bib-0024] LeLaurin, J. H. , & Shorr, R. I. (2019). Preventing falls in hospitalized patients: State of the science. Clinics in Geriatric Medicine, 35(2), 273–283. 10.1016/j.cger.2019.01.007 30929888 PMC6446937

[nop21975-bib-0025] Li, X. , & Fawcett, T. N. (2014). Clinical decision making on the use of physical restraint in intensive care units. International Journal of Nursing Sciences, 1(4), 446–450. 10.1016/j.ijnss.2014.09.003

[nop21975-bib-0026] Martin, B. , & Mathisen, L. (2005). Use of physical restraints in adult critical care: A bicultural study. American Journal of Critical Care, 14(2), 133–142. 15728955

[nop21975-bib-0027] Nakanishi, M. , Okumura, Y. , & Ogawa, A. (2018). Physical restraint to patients with dementia in acute physical care settings: Effect of the financial incentive to acute care hospitals. International Psychogeriatrics, 30(7), 991–1000. 10.1017/S104161021700240X 29122058

[nop21975-bib-0028] Perez, D. , Peters, K. , Wilkes, L. , & Murphy, G. (2019). Physical restraints in intensive care‐an integrative review. Australian Critical Care, 32(2), 165–174. 10.1016/j.aucc.2017.12.089 29559190

[nop21975-bib-0029] Registered Nurses' Association of Ontario [RNAO] . (2012). Promoting safety: Alternative approaches to the use of restraints . https://rnao.ca/bpg/guidelines/promoting‐safety‐alternative‐approaches‐use‐restraints

[nop21975-bib-0030] Scheepmans, K. , Dierckx de Casterlé, B. , Paquay, L. , Van Gansbeke, H. , & Milisen, K. (2020). Reducing physical restraints by older adults in home care: Development of an evidence‐based guideline. BMC Geriatrics, 20(1), 169. 10.1186/s12877-020-1499-y 32380959 PMC7204038

[nop21975-bib-0031] Siegrist‐Dreier, S. , Barbezat, I. , Thomann, S. , Richter, D. , Hahn, S. , & Schmitt, K.‐U. (2022). Restraining patients in acute care hospitals—A qualitative study on the experiences of healthcare staff. Nursing Open, 9(2), 1311–1321. 10.1002/nop2.1175 35088948 PMC8859077

[nop21975-bib-0038] Spennato, U. , Lerjen, N. , Siegwart, J. , Mueller, B. , Schuetz, P. , Koch, D. , & Struja, T. (2023). Prevalence, risk factors and outcomes associated with physical restraint in acute medical Inpatients over 4 years‐a retrospective cohort study. Geriatrics (Basel), 8(1). 10.3390/geriatrics8010015 PMC9957493 36826357

[nop21975-bib-0032] Swiss Academy of Medical Sciences . (2015). Coercive measures in medicine. Medical‐ethical guidelines . https://www.sams.ch/en/Publications/Medical‐ethical‐Guidelines.html

[nop21975-bib-0033] Thomann, S. , Zwakhalen, S. , Richter, D. , Bauer, S. , & Hahn, S. (2021). Restraint use in the acute‐care hospital setting: A cross‐sectional multi‐Centre study. International Journal of Nursing Studies, 114, 103807. 10.1016/j.ijnurstu.2020.103807 33217663

[nop21975-bib-0034] Thomann, S. , Zwakhalen, S. , Siegrist‐Dreier, S. , & Hahn, S. (2022). Restraint practice in the somatic acute care hospital: A participant observation study. Journal of Clinical Nursing, 32(11–12), 2603–2615. 10.1111/jocn.16322 35451093

[nop21975-bib-0041] Tong, A. , Sainsbury, P. , & Craig, J. (2007). Consolidated criteria for reporting qualitative research (COREQ): A 32‐item checklist for interviews and focus groups. International Journal for Quality in Health Care, 19(6), 349–357. 10.1093/intqhc/mzm042. 17872937

[nop21975-bib-0035] VERBI Software . (2019). MAXQDA 2019 [computer software]. VERBI Software Retrieved from maxqda.com

[nop21975-bib-0036] Warlan, H. , & Howland, L. (2015). Posttraumatic stress syndrome associated with stays in the intensive care unit: Importance of nurses' involvement. Critical Care Nurse, 35(3), 44–52; quiz 54. 10.4037/ccn2015758 26033100

[nop21975-bib-0037] Wong, A. H. , Ray, J. M. , Rosenberg, A. , Crispino, L. , Parker, J. , McVaney, C. , Iennaco, J. D. , Bernstein, S. L. , & Pavlo, A. J. (2020). Experiences of individuals who were physically restrained in the emergency department. JAMA Network Open, 3(1), e1919381. 10.1001/jamanetworkopen.2019.19381 31977058 PMC6991263

